# Forcecardiography: A Novel Technique to Measure Heart Mechanical Vibrations onto the Chest Wall

**DOI:** 10.3390/s20143885

**Published:** 2020-07-13

**Authors:** Emilio Andreozzi, Antonio Fratini, Daniele Esposito, Ganesh Naik, Caitlin Polley, Gaetano D. Gargiulo, Paolo Bifulco

**Affiliations:** 1 Department of Electrical Engineering and Information Technologies, University of Naples Federico II, Via Claudio, 21 80125 Napoli, Italy; emilio.andreozzi@unina.it (E.A.); daniele.esposito@unina.it (D.E.); 2 Istituti Clinici Scientifici Maugeri S.p.A.—Società benefit, Via S. Maugeri, 10 27100 Pavia, Italy; 3 Biomedical Engineering, School of Life and Health Sciences, Aston University, Birmingham B4 7ET, UK; a.fratini@aston.ac.uk; 4 The MARCS Institute, Western Sydney University, Penrith NSW 2751, Australia; ganesh.naik@westernsydney.edu.au (G.N.); g.gargiulo@uws.edu.au (G.D.G.); 5 School of Computing, Engineering, and Mathematics, Western Sydney University, Penrith NSW 2747, Australia; caitlin.polley@westernsydney.edu.au

**Keywords:** seismocardiography, force-sensitive resistor, non-invasive sensor, cardiac monitoring, cardiac function

## Abstract

This paper presents forcecardiography (FCG), a novel technique to measure local, cardiac-induced vibrations onto the chest wall. Since the 19th century, several techniques have been proposed to detect the mechanical vibrations caused by cardiovascular activity, the great part of which was abandoned due to the cumbersome instrumentation involved. The recent availability of unobtrusive sensors rejuvenated the research field with the most currently established technique being seismocardiography (SCG). SCG is performed by placing accelerometers onto the subject’s chest and provides information on major events of the cardiac cycle. The proposed FCG measures the cardiac-induced vibrations via force sensors placed onto the subject’s chest and provides signals with a richer informational content as compared to SCG. The two techniques were compared by analysing simultaneous recordings acquired by means of a force sensor, an accelerometer and an electrocardiograph (ECG). The force sensor and the accelerometer were rigidly fixed to each other and fastened onto the xiphoid process with a belt. The high-frequency (HF) components of FCG and SCG were highly comparable (r > 0.95) although lagged. The lag was estimated by cross-correlation and resulted in about tens of milliseconds. An additional, large, low-frequency (LF) component, associated with ventricular volume variations, was observed in FCG, while not being visible in SCG. The encouraging results of this feasibility study suggest that FCG is not only able to acquire similar information as SCG, but it also provides additional information on ventricular contraction. Further analyses are foreseen to confirm the advantages of FCG as a technique to improve the scope and significance of pervasive cardiac monitoring.

## 1. Introduction

Body vibrations induced by the activity of the cardiovascular system have been recorded since the second half of the 19th century [1,2]. Several different techniques and apparatuses were proposed as non-invasive ways for the investigation of cardiac mechanics [3,4]. The initial complications, due to the cumbersome instrumentation and the lack of reliable annotation standards, made them almost disappear from scientific research, in favour of more appealing techniques [5]. As soon as the technological advancements provided modern unobtrusive sensors, one the old-fashioned techniques came back in vogue—the seismocardiography (SCG) [5,6,7].

SCG is a non-invasive technique for the measurement of local cardiac-induced vibrations of the chest wall that earned growing interest due to the recent availability of small and lightweight accelerometers [5,6,7,8,9,10,11,12,13,14,15,16,17,18]. These are indeed the most common sensors used to date to acquire kinematic measurements onto the chest wall. SCG offers the possibility to investigate and monitor the mechanical behaviour of the heart and, therefore, it can provide additional information to the electrocardiograph (ECG) [5]. According to the literature, many events, such as cardiac valves opening and closing, isovolumic contraction, cardiac filling and blood injection, relate well to peaks and valleys of the SCG signals [6,8]. Hence, it is possible to estimate time intervals and velocities of physiological relevance, which give important insights into the mechanics of the beating heart (such as left ventricular ejection time, rapid diastolic filling time, isovolumic contraction and relaxation times [9,10], left ventricular lateral wall and septal wall contraction peak velocities, trans-aortic and trans-pulmonary peak flows [11]). The latest studies on methods for the detection of local heart mechanical vibrations of the chest wall have also used gyroscopes [19,20,21], laser Doppler vibrometers [22], microwave Doppler radars [23,24], airborne ultrasound surface motion cameras [25] and polyvinylidene fluoride (PVDF) piezoelectric sensors [26].

Recently, a novel sensor was presented, which was able to monitor the small mechanical vibrations of a contracting muscle (referred to as mechanomyogram (MMG)) by means of a dedicated force sensor, in place of microphones and accelerometers [27]. Its features led to the investigation of its potential use for the detection of mechanical vibrations on different body parts, particularly onto the chest wall.

A novel technique to measure the local mechanical vibrations induced on a patient’s chest wall by the beating heart is proposed. It is based on force sensors and therefore named forcecardiography (FCG). FCG is part of a more complex signal and device for which some of the authors have filed a patent (AUPRV 2019903937) scheduled to appear for public release in late 2020. The new FCG signals were compared to the well-established accelerometric SCG recordings, both acquired on a standard chest location (xiphoid process) in parallel to a single-lead ECG. An additional, large, low-frequency (LF) signal associated with ventricular volume variations showed up in the FCG, while not being visible in the SCG. The SCG and the high-frequency (HF) components of the FCG signal turned out to be very similar but lagged. The mechanical impedances of the tissues and sensors, which exhibit a viscoelastic behaviour [28], strongly influence the relationship between force and acceleration, by introducing attenuations and phase shifts that vary with frequency. The results suggest that FCG is able to acquire practically the same information of SCG, while providing new additional information that can improve pervasive cardiac monitoring.

## 2. Materials and Methods

### 2.1. Forcecardiography Sensor

**Figure 1:**
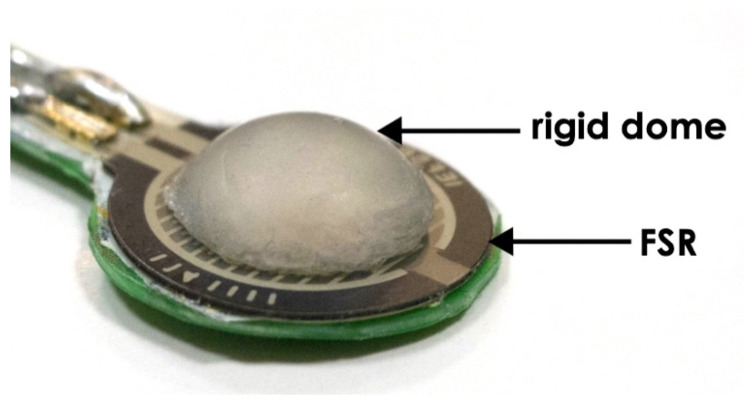
Force-sensitive resistor (FSR)-based forcecardiography (FCG) sensor.

The sensor used for FCG measurements, presented in [27], is shown in Figure 1; it includes the force-sensitive resistor (FSR) Interlink FSR402 Short (Interlink Electronics Inc., Camarillo, CA, USA), with a rigid dome fixed on its active area. The FSR is conditioned by means of a transimpedance amplifier, which provides an output proportional to the force and minimizes sensor drift by keeping the voltage across the FSR at a constant value [27]. The transimpedance amplifier is followed by an analog active high-pass filter (3 dB cut-off frequency 0.16 Hz), which removes the DC component, thus weakening the effect of sensor drift. The FCG sensor was calibrated before measurements, in order to obtain the transduction coefficient from voltage to force [27]. In particular, different weights were applied onto the FSR-based sensor and measured together with the resulting voltages. Linear regression of the experimental data provided the actual sensitivity of the FSR-based sensor (see [27] for further details).

### 2.2. Measurement Setup and Procedure

In addition to the FCG sensor, a Freescale MMA7361 analog triaxial accelerometer (Freescale Semiconductor - NXP Semiconductors, Eindhoven, The Netherlands) was used to acquire the dorso-ventral SCG signal and a WelchAllyn Propaq^®^ Encore monitor (Welch Allyn Inc., New York, NY, USA) was used to record the ECG lead II. The accelerometer output was processed by the same analog active high-pass filter (3 dB cut-off frequency 0.16 Hz) used for the FCG sensor to remove the DC component (gravitational acceleration).

**Figure 2:**
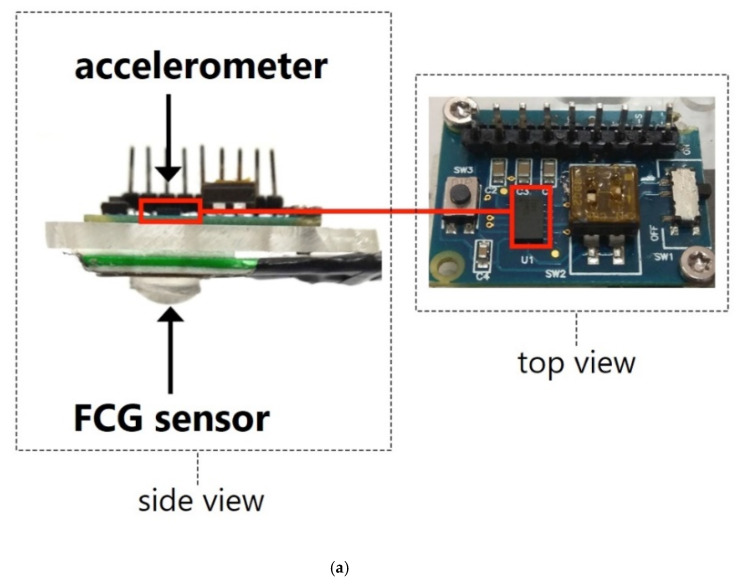

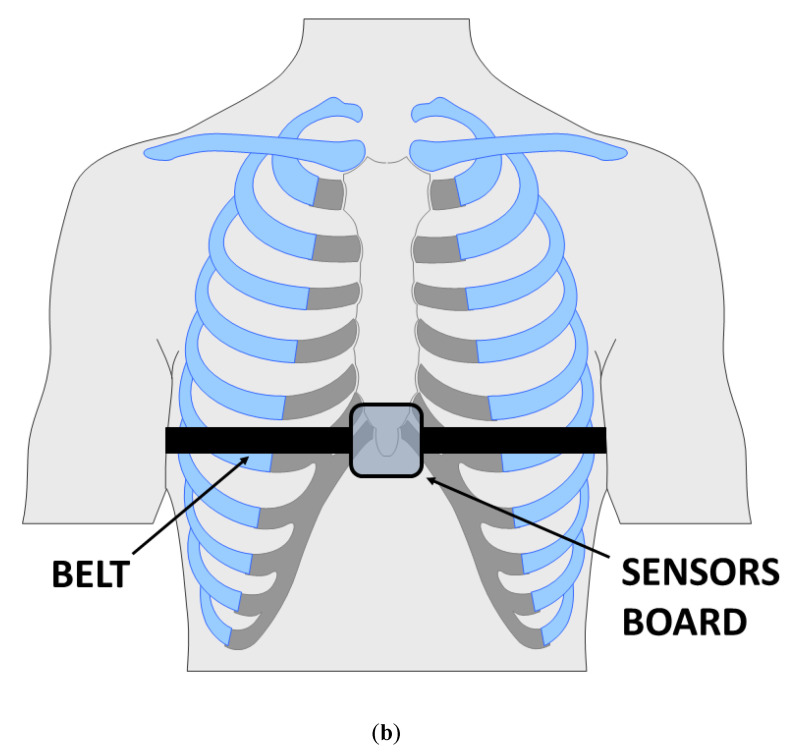
(**a**) Side and top views of the sensors board; (**b**) sensors board positioning.

The FCG sensor and the accelerometer were firmly mounted on a plexiglass rigid board (Figure 2a) in order to make them integral, as this would help in obtaining a reliable comparison between their measurements. This sensors board was then fastened onto the subjects’ chest wall, exactly onto the xiphoid process, by means of a belt (Figure 2b).

All the measurements were performed on some of the authors (5 male subjects aged 41.0 ± 10.4 years and body mass index of 26.6 ± 2.44 kg/m^2^), lying on a comfortable deck chair while holding their breath after inhalation, in order to limit motion artifacts and respiration-induced variability of the SCG signals’ shape [7]. The signals from the FCG sensor, accelerometer and ECG monitor were synchronously recorded with a sampling frequency of 10 kHz, 14-bit precision via a National Instrument NI-USB6009 DAQ board (National Instruments Corp., Austin, TX, USA).

### 2.3. Signal Processing

The accelerometer signal was reversed in amplitude as its positive acceleration direction was opposite to the positive force direction of the FCG sensor. Both FCG and SCG signals were filtered in order to separate the low-frequency and high-frequency informational content. The low-frequency and high-frequency components of FCG and SCG were extracted by means of a 0.5–5 Hz band-pass filter and a 10–20 Hz band-pass filter, respectively.

The ECG-triggered ensemble averages (synchronised with R peaks) of the ECG and the low-frequency and high-frequency components of FCG and SCG were computed on 36 consecutive heartbeats. The R-peaks were located with the algorithm described in [29]. This is a common practice in SCG studies, which is aimed at filtering out inter-beat variations and extracting common features [6,7,8,14,15,16,19,20,21,24]. For each signal, segments of 700 ms (from 180 ms before R-peaks to 520 ms after) were considered for the analysis.

As reported in the Results section, the high-frequency components of the FCG and SCG signals appeared lagged. Hence, the lag between the two ensemble averages was estimated by locating the maximum of their cross-correlation function. Pearson’s correlation coefficient was used as a similarity metric to separately compare the ensemble averages of corresponding low-frequency components and high-frequency components of the FCG and SCG signals.

To assess the correlation of FCG with the cardiac cycle, a beat-by-beat comparison of FCG and ECG signals was carried out by comparing the inter-beat intervals estimated from ECG, LF-FCG and HF-FCG. In LF-FCG signals, the largest negative peak after each ECG R peak was considered as a fiducial point and located by searching for the absolute minimum in a window of 520 ms after each R peak. In HF-FCG signals, the first positive peak after each ECG R peak was considered as a fiducial point and located by searching for the first positive peak in a window of 520 ms after each R peak, via the MATLAB function “findpeaks”. The inter-beat intervals estimated from LF-FCG and HF-FCG were compared against those estimated from the ECG by means of correlation and Bland–Altman analyses, which were carried out via the MATLAB function “bland-altman-and-correlation-plot” [30].

## 3. Results

The static pressure exerted on the FSR-based sensor (i.e., the pressure resulting from the tightening of the belt around the thorax) was about 50 g/cm^2^, on average, considering all measurements.

**Figure 3:**
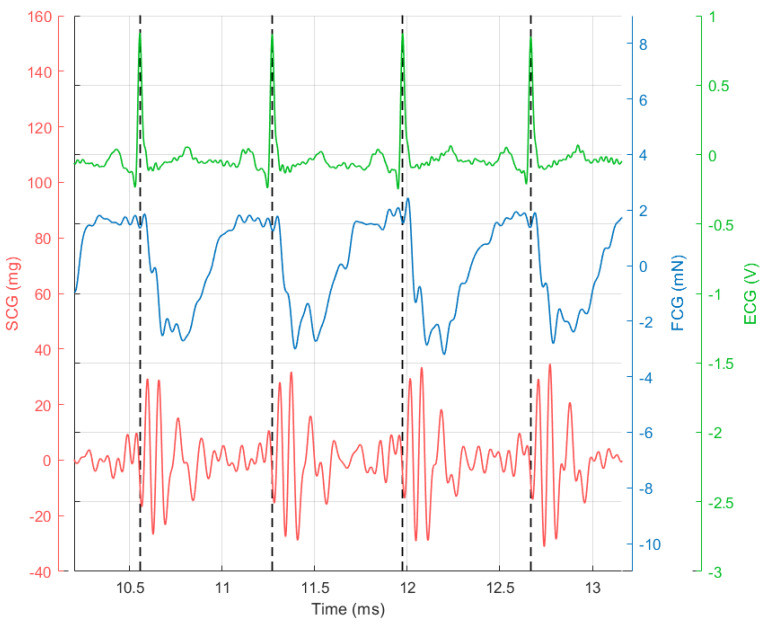
Subject #2 dataset: electrocardiograph (ECG) is shown in green and scaled in mV, FCG is shown in blue and scaled in mN and seismocardiography (SCG) (dorso-ventral acceleration) is shown in red and scaled in mg. The R-peaks time references correspond to the black dashed lines.

The ECG, along with FCG and dorso-ventral acceleration (SCG) signals filtered in the 0.5–20 Hz frequency band, are reported in Figure 3 (four consecutive heartbeats).

**Figure 4:**
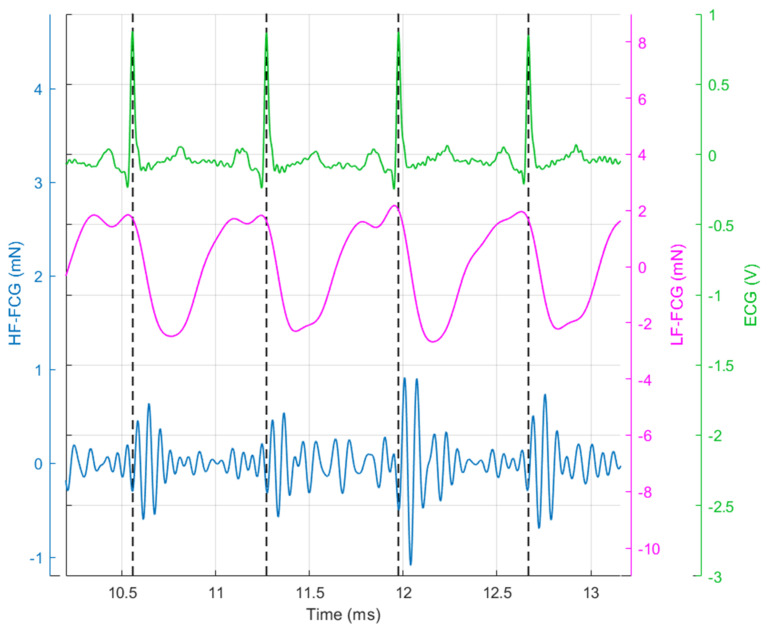
Subject #2 dataset: ECG is shown in green and scaled in mV, low-frequency (LF)-FCG is shown in purple and scaled in mN and high-frequency (HF)-FCG is shown in blue and scaled in mN.

It can be clearly seen that the FCG signal (blue line) is characterised by the superimposition of a large low-frequency component (i.e., the large negative peaks that occur after each QRS complex of the ECG) and a smaller high-frequency component (i.e., the pattern of small oscillations that are superimposed on a single large negative peak between two heartbeats). In Figure 4, the low-frequency and high-frequency components of the FCG signal (referred to as LF-FCG and HF-FCG, respectively) are depicted.

**Figure 5:**
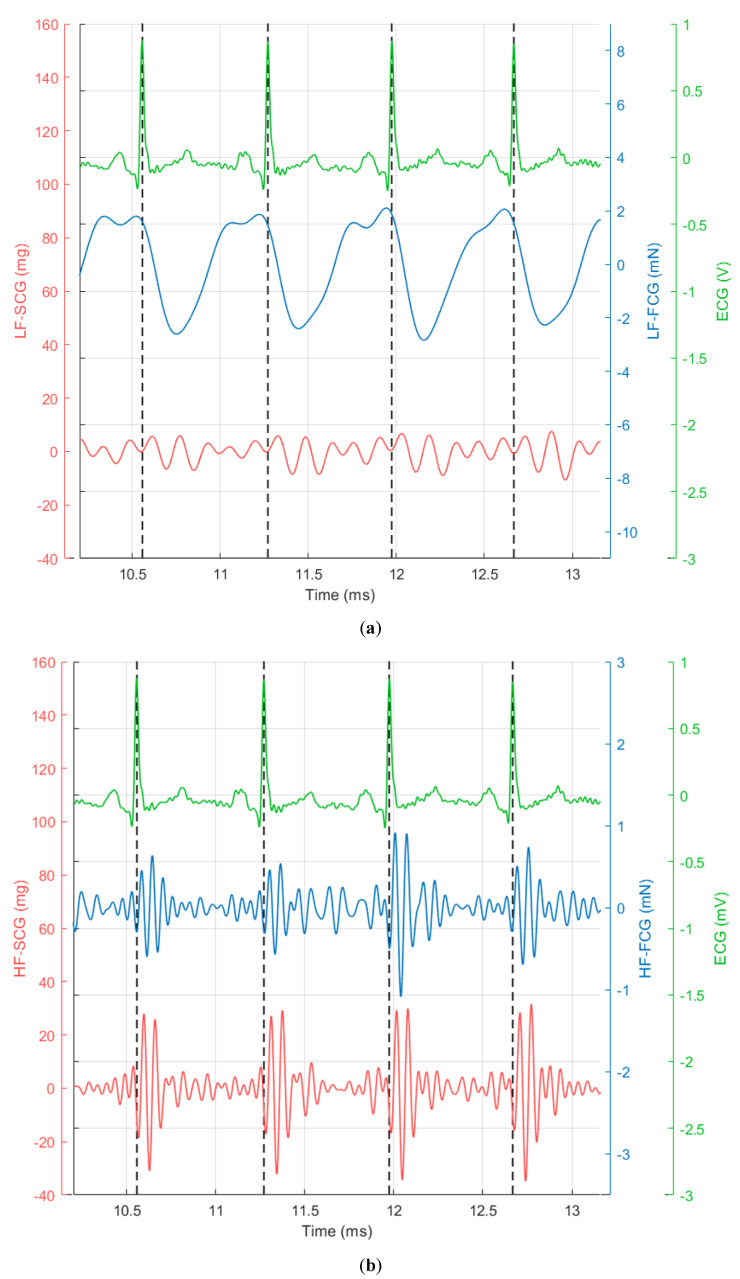
Subject #2 dataset: (**a**) ECG with low-frequency components of FCG and SCG; (**b**) ECG with high-frequency components of FCG and SCG. ECG is shown in green and scaled in mV, FCG is shown in blue and scaled in mN, SCG (dorso-ventral acceleration) is shown in red and scaled in mg. The R-peaks time references correspond to the black dashed lines.

It can be observed that the HF-FCG component resembles the HF-SCG, and it is even more evident from Figure 5, where one can observe that HF-FCG and HF-SCG share similar morphology, with the latter being consistently lagged with respect to the former. Instead, the low-frequency component cannot be appreciated in the SCG signal, which presents only higher frequency oscillations.

**Figure 6:**
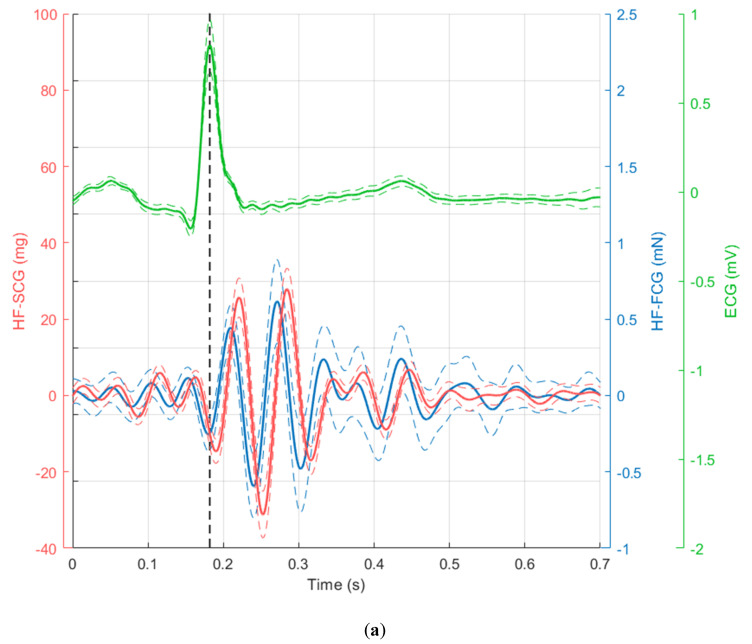

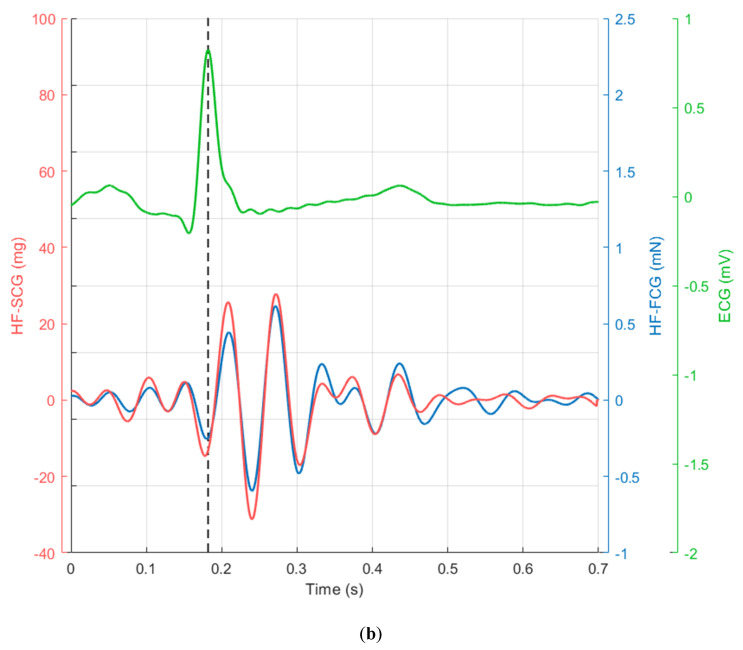
Subject #2 dataset. (**a**) ECG, HF-FCG and HF-SCG ensemble averages on 36 normal heartbeats (solid lines) with relative standard deviation (SD) intervals (dashed lines); (**b**) ensemble averages of ECG, HF-FCG and lag-compensated HF-SCG.

The ensemble averages of the ECG, HF-FCG and HF-SCG signals are shown in Figure 6a, along with their standard deviation (SD) intervals. The lag between the HF-FCG and HF-SCG ensemble averages in Figure 6a was equal to 12.3 milliseconds. This value was used to realign the HF-FCG and HF-SCG ensemble averages (depicted in Figure 6b). A quantitative measure of their similarity was provided by their Pearson’s correlation coefficient, which was higher than 0.95 (R^2^ > 0.90, *p* < 0.05). To further assess the lack of correlation between LF-FCG and the corresponding low-frequency component of SCG (i.e., filtered in the 0.5–5 Hz frequency band), the same procedure was applied to obtain their Pearson’s correlation coefficient, which turned out to be lower than 0.32 (R^2^ < 0.10, *p* < 0.05).

**Figure 7:**
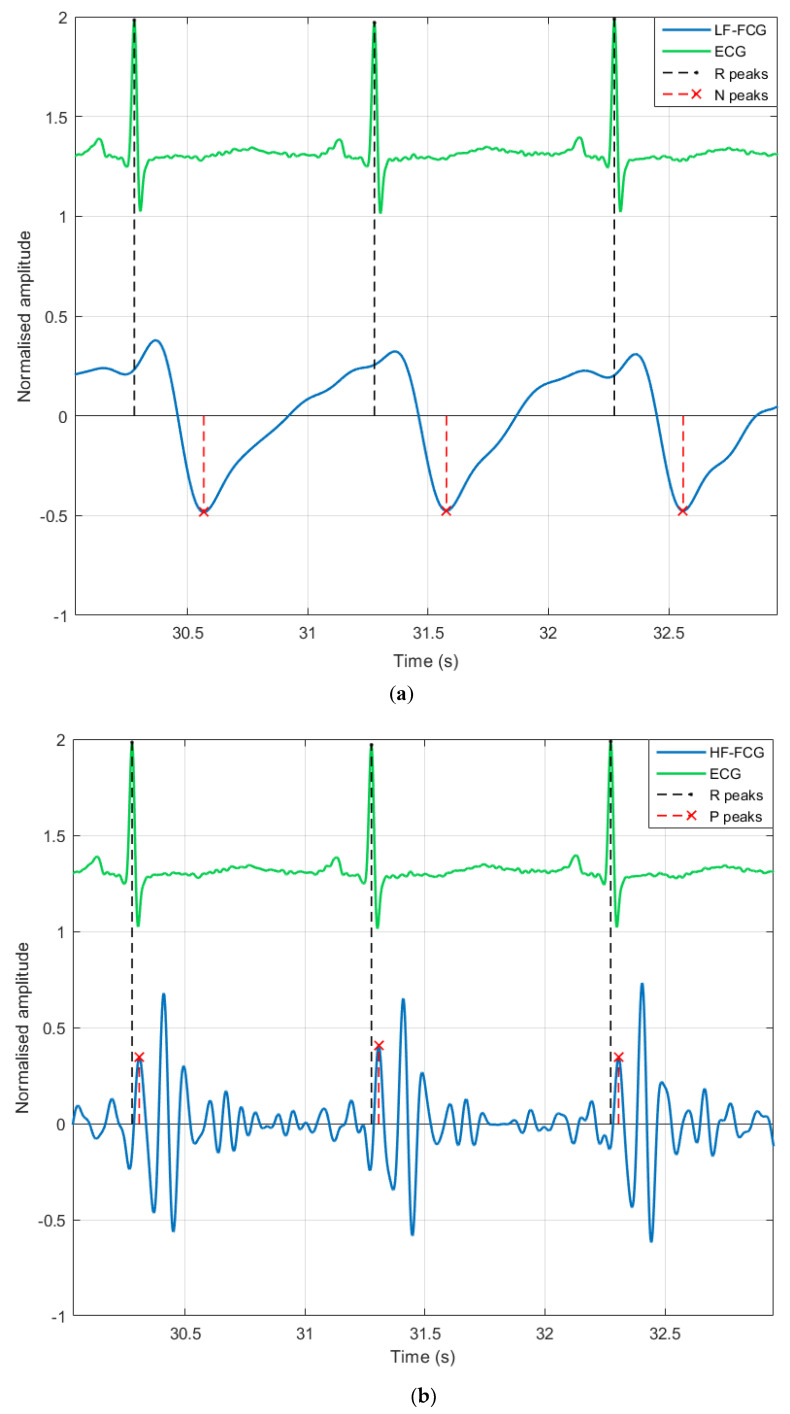
Subject #5 dataset. (**a**) Excerpt of ECG and LF-FCG signals (3 consecutive heartbeats) showing fiducial points located on LF-FCG; (**b**) excerpt of ECG and HF-FCG signals (same 3 consecutive heartbeats) showing fiducial points located on HF-FCG.

Figure 7 shows an example of fiducial point localization on LF-FCG and HF-FCG for three consecutive heartbeats, along with the related ECG R peaks. Small tracts corrupted by motion artifacts were excluded. A total of 223 inter-beat intervals were extracted from ECG, LF-FCG and HF-FCG and compared by means of correlation and Bland–Altman analyses.

**Figure 8:**
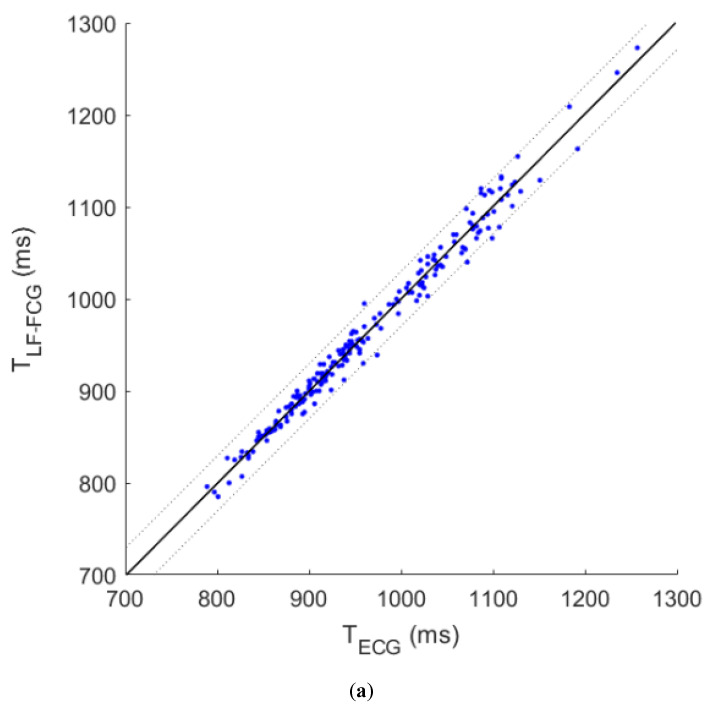

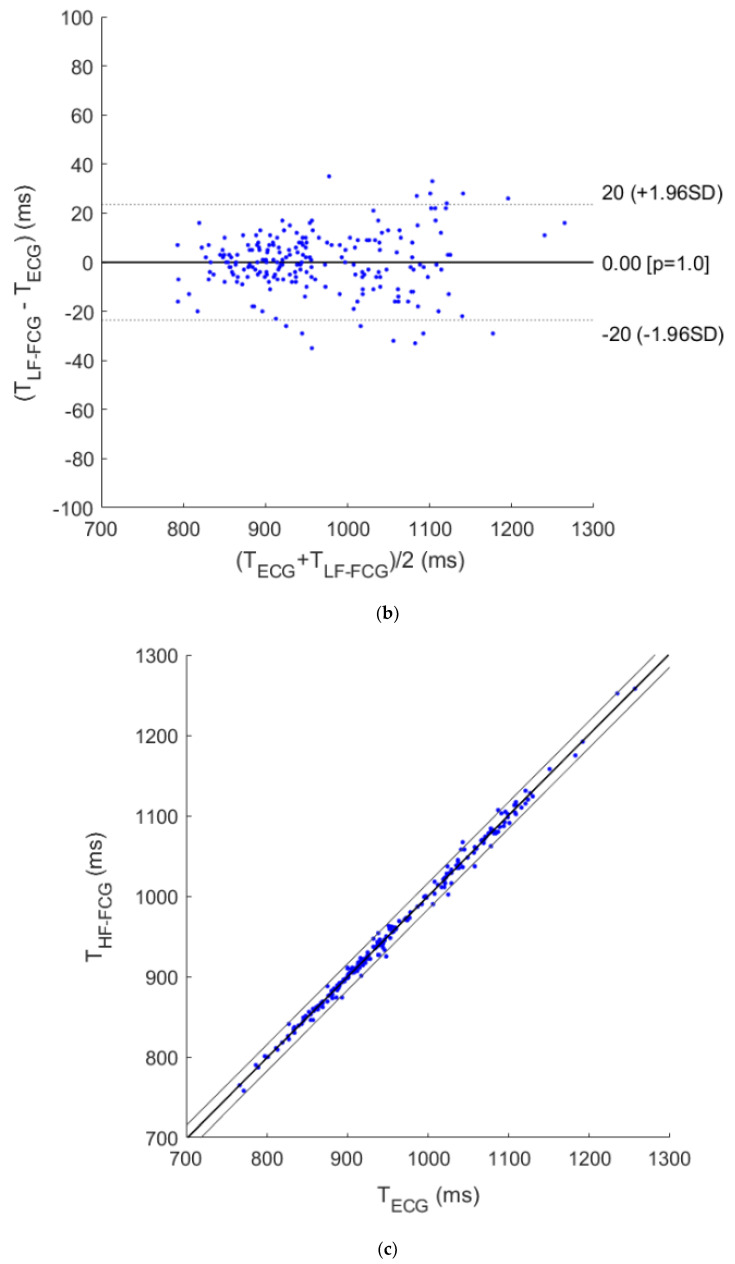

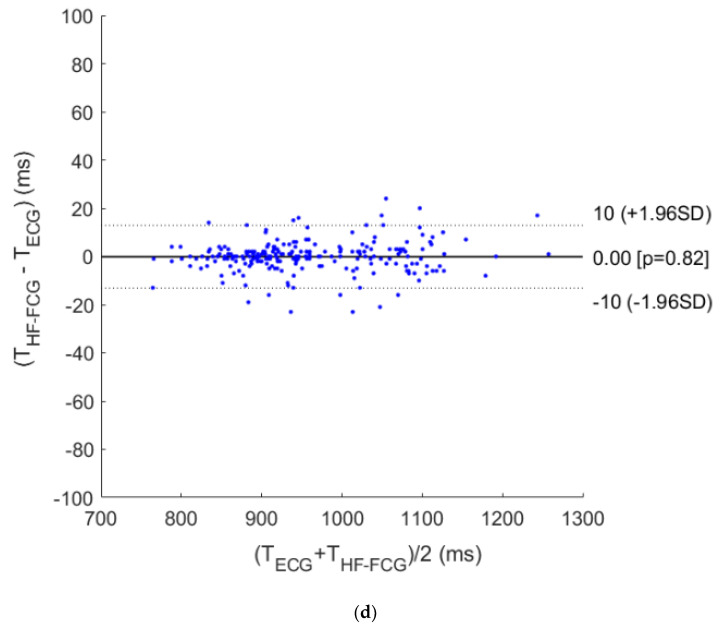
(**a**,**b**) Results of correlation and Bland–Altman analyses for inter-beat intervals estimated from ECG and LF-FCG; (**c**,**d**) Results of correlation and Bland–Altman analyses for inter-beat intervals estimated from ECG and HF-FCG. Bias significances were determined via one-paired *t*-test and the resulting *p*-values were reported on the plots.

The results of correlation and Bland–Altman analyses are depicted in Figure 8. The correlation analysis reported a slope of 1.004 and intercept of −0.004 for both LF-FCG and HF-FCG, with R^2^ values of 0.984 for the former and 0.995 for the latter. The Bland–Altman analysis reported a null bias for both LF-FCG and HF-FCG, with limits of agreement of 23.5 ms and 13.0 ms, respectively.

## 4. Discussion

A novel technique for the measurement of local cardiac-induced mechanical vibrations of the chest wall was presented. It was named forcecardiography, since it employs dedicated force sensors as opposed to the well-established accelerometric seismocardiography.

The two techniques were compared by recording simultaneous signals from an FCG sensor and an accelerometer, firmly mounted on the same rigid board and fastened with a belt onto the xiphoid process. An ECG lead was concurrently acquired to provide a precise time reference.

The FCG was represented by the superimposition of a large low-frequency component and a smaller high-frequency component. The low-frequency component was characterised by large negative force peaks, which correspond to forces directed inward and seem to be associated with the ventricular emptying. No such component was found in the SCG signal, which, therefore, cannot provide information about this aspect of cardiac mechanics. The high-frequency component was characterised by a pattern of small oscillations that resemble the SCG.

Therefore, their similarity was quantitatively assessed by evaluating their Pearson’s correlation coefficient. To this aim, the ensemble averages of HF-FCG and HF-SCG signals were computed, in order to filter out inter-beat variations, which could had undermined the results.

A lag in the order of tens of milliseconds was observed between the ensemble averages of HF-FCG and HF-SCG, with the latter being consistently lagged with respect to the former. Certainly, the relationship between force and acceleration is quite complex and may introduce different attenuations and phase shifts to different spectral components, depending on their frequency. Indeed, the beating heart is a source of mechanical force that propagates outward through different human tissues, which exhibit a viscoelastic behaviour [28]. Therefore, it is clear that the relationship between force and acceleration at the outmost tissues strongly depends on their mechanical properties, which are described by their mechanical impedances. However, a proper characterisation of this relationship is beyond the scope of this article.

The Pearson’s correlation coefficient of HF-FCG and HF-SCG ensemble averages scored values greater than 0.95 (R^2^ > 0.90, *p* < 0.05), thus providing a quantitative proof of their similarity. Such high similarity emerging from the preliminary results suggests that HF-FCG provides practically the same information as that of HF-SCG.

Moreover, correlation and Bland–Altman analyses were carried out on the inter-beat intervals estimated with ECG, LF-FCG and HF-FCG. The results obtained from these analyses (unit slope and null intercept with R^2^ > 0.98; null bias with limits of agreement up to 24 ms) confirmed that both LF-FCG and HF-FCG are highly correlated with heart contractions.

In conclusion, the proposed FCG technique allows to acquire signals with a richer informational content, as compared to the well-established accelerometric SCG, and so it provides new additional information that could improve the investigation and comprehension of the mechanical behaviour of the beating heart. Further analyses are foreseen to confirm the advantages of FCG (e.g., in clinical applications as that described in [31]) and to assess the LF-FCG relationship with cardiac mechanics, also by comparing it with simultaneous echocardiographic recordings [32]. This also paves the way for further studies about the use of force sensors in pervasive patient monitoring [33,34]. As an example, textile force sensors can be easily embedded in washable smart garments [35,36], as opposed to accelerometers that would need to be removed as they are not waterproof. The use of multiple force sensors [37] could provide new information on the propagation and distribution of heart mechanical vibrations onto the chest wall.
